# Hepatitis B Viral Protein HBx and the Molecular Mechanisms Modulating the Hallmarks of Hepatocellular Carcinoma: A Comprehensive Review

**DOI:** 10.3390/cells11040741

**Published:** 2022-02-21

**Authors:** Enakshi Sivasudhan, Neil Blake, Zhiliang Lu, Jia Meng, Rong Rong

**Affiliations:** 1 Department of Biological Sciences, Xi’an Jiaotong-Liverpool University, Suzhou 215123, China; e.sivasudhan19@student.xjtlu.edu.cn (E.S.); zhiliang.lu@xjtlu.edu.cn (Z.L.); jia.meng@xjtlu.edu.cn (J.M.); 2 Department of Clinical Infection, Microbiology and Immunology, Institute of Infection, Veterinary and Ecological Sciences, University of Liverpool, Liverpool L69 7BE, UK; nwblake@liverpool.ac.uk; 3 Institute of Integrative Biology, University of Liverpool, Liverpool L69 7ZB, UK

**Keywords:** hepatitis B virus, HBx protein, hepatocellular carcinoma, cancer hallmarks, therapeutics

## Abstract

With 296 million cases estimated worldwide, chronic hepatitis B virus (HBV) infection is the most common risk factor for hepatocellular carcinoma (HCC). HBV-encoded oncogene X protein (HBx), a key multifunctional regulatory protein, drives viral replication and interferes with several cellular signalling pathways that drive virus-associated hepatocarcinogenesis. This review article provides a comprehensive overview of the role of HBx in modulating the various hallmarks of HCC by supporting tumour initiation, progression, invasion and metastasis. Understanding HBx-mediated dimensions of complexity in driving liver malignancies could provide the key to unlocking novel and repurposed combinatorial therapies to combat HCC.

## 1. Introduction

Despite the availability of hepatitis B virus (HBV) vaccines, the worldwide incidence of hepatitis B cases remain to be an estimated 296 million, with over 1.5 million new infections occurring annually [1]. Prolonged chronic inflammation and tissue damage associated with HBV often lead to fibrosis/cirrhosis and eventually hepatocellular carcinoma (HCC), the most common form of liver cancer [2]. According to the latest epidemiological data from Globocan 2020 report, newly diagnosed liver cancer cases account for over 900,000 cancer cases annually (4.7% of all cancers) and there are approximately 830,000 deaths linked to HCC annually [3]. Geographic variations associated with HBV infection show 70–80% hepatitis B surface antigen (HBsAg) seroprevalence in South East Asia and Sub-Saharan Africa, linked to high levels of HBV driven HCC incidence, whereas less than 2% HBsAg seroprevalence is observed in Western Europe, America and Australia with a similar low level of HBV-driven HCC [4].

HBV is a small DNA virus, a prototype of *Hepadnaviridae* family of viruses, with a 3.2 kilobases circular and partially double stranded genome. During replication, the partially dsDNA genome is gap repaired and transcribed into a full length pre-genomic RNA which is subsequently reverse transcribed by a viral polymerase with RT activity. The genome encodes four overlapping open reading frames (ORF) encoding envelope protein (pre-S1/pre-S2), core protein (pre-C/C), viral polymerase and X protein (HBx) [5]. While the exact molecular mechanisms driving HBV-induced HCC have been poorly defined, it has been speculated that virus-host genome integration, prolonged inflammation aided host immune response and cellular signal transduction pathways altered by the viral regulatory protein HBx could play a crucial role in the progression of liver tumorigenesis [6,7,8].

HBx gene, the smallest ORF of the HBV genome, encodes a 154-amino acid regulatory protein of molecular weight 17 kDa. It is found in all mammalian *Hepadnaviruses*, termed *Orthohepadnaviruses*, but interestingly not in avian species which are infected by *Avihepadnaviruses*. HBx proteins from different species contain conserved regions, helical structures in the amino- and carboxy-terminal as well as a coil-to-coil motif with several regions with known functional domains [9]. Attempts at crystallizing the protein have been proven futile, so no detailed structural information is available. HBx forms homodimers via disulfide bonds and acetylation [10]. It received its name, X protein, due to the lack of sequence homology to any existing protein. Intracellular localization studies based on HBV patient-derived liver biopsies show that HBx predominantly accumulates in the cytoplasm when highly expressed, whereas low expression leads to localization primarily in the nucleus [11,12].

While the precise functions of HBx are not fully understood, it has been proposed that HBx is multifunctional owing to its negative regulatory/anti-apoptotic N terminal and transactivator C terminal in addition to being the only regulatory protein encoded by the virus. HBx is key to driving HBV viral infection via governing cellular and viral promoters and enhancers, driving promiscuous transactivation, through protein–protein interactions and, without directly binding to DNA [13,14]. Given its primary localization in the cytoplasm, HBx is able to modulate several signal transduction pathways such as mitogen-activated protein kinase (MAPK), nuclear factor kappa-light-chain-enhancer of activated B cells (NF-kB), rat sarcoma virus (Ras), rapidly accelerated fibrosarcoma (Raf), janus kinase-signal transducer and activator of transcription (JAK-STAT), focal adhesion kinase (FAK) and kinase C signalling cascades. [15,16]. Furthermore, HBx has also been implicated in cell cycle regulation, calcium signalling, DNA repair and regulation of apoptosis [17]. The potential role of HBx in hepatocarginogenesis was apparent with the development of HCC in the natural hosts of HBx encoding Orthohepadnaviruses, woodchucks, squirrel monkeys and humans, whereas there is no evidence of liver tumourigenesis for infection with Avihepadnaviruses which lack the HBx protein [18]. In vitro studies have shown that prolonged expression of HBx induces cellular transformation of rodent hepatocytes, while the presence of integrated HBx gene in the chromosomal DNA has been frequently detected in patients diagnosed with HCC [19,20].

**Figure 1:**
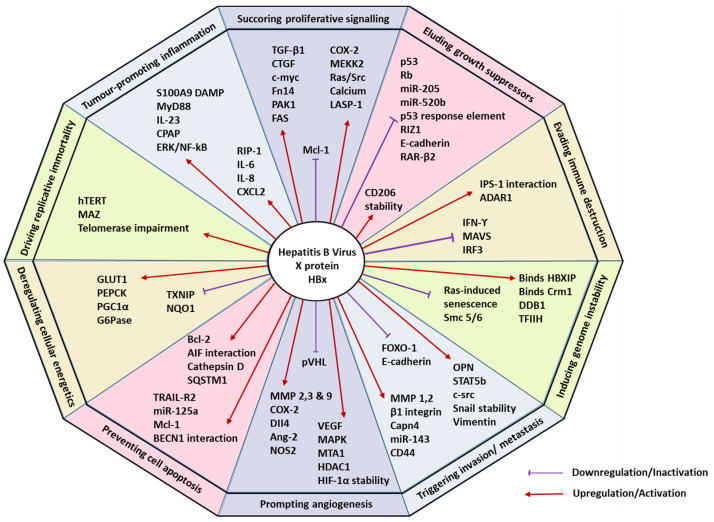
Hallmarks of hepatocellular carcinoma modulated by hepatitis B virus X protein (HBx). Numerous liver tumorigenesis-driving hallmarks influence the downstream cellular mechanisms by sustaining proliferative signalling, eluding growth suppressors, evading immune destruction, facilitating replicative immortality, aiding in tumour-promoting inflammation, triggering invasion and metastasis, prompting angiogenesis, inducing genome instability, preventing cell apoptosis, and deregulating cellular energetics.

The multifunctional nature of HBx implies its involvement in altering several cellular mechanisms by hijacking the host cell homeostasis and provoking tumorigenic traits capable of inducing HCC. Such complex and distinct traits that trigger tumorigenesis and metastatic propagation, in general, have been organized into 10 major hallmarks of cancer by Hanahan and Weinberg [21,22]. These hallmarks are sustaining proliferative signalling, eluding growth suppressors, evading immune destruction, facilitating replicative immortality, aiding in tumour-promoting inflammation, triggering invasion and metastasis, prompting angiogenesis, inducing genomic instability, preventing cell apoptosis, and deregulating cellular energetics [22]. This article discusses the involvement of HBx in modulating the different hallmarks of HCC, highlighting the potential therapeutic implications. Table 1 and Figure 1 provide a summary and overview of HBx-mediated hallmarks of HCC.

## 2. Sustaining Proliferative Signalling

According to Hanahan and Weinberg, cancer cells endure proliferative signalling by enabling growth factor expression, stimulating non-malignant cells within the tumour-associated stroma, upregulating receptor protein levels in cancer cells and altering the structures of receptor molecules while activating downstream mitogenic signalling pathways linked to these receptors [22]. Conceivably, the most significant function of HBx is its ability to promote cell proliferation in hepatocarcinogenesis. For example, HBx has been proposed to activate stellate cells in fibrosis and elevate expression of transforming-growth factor β1 (TGF-β1) and connective tissue growth factor (CTGF) thus promoting cellular transformation [23].

HBx-transgenic liver cancer mouse model studying pre-neoplasm showed a five-fold elevated expression of c-myc, a multifunctional transcription factor which promotes cell proliferation, while an In vitro study established a strong correlation between HBx and c-myc which abetted ribosome biogenesis and cellular transformation [24,25]. Another In vitro study involving HepG2 hepatoma cells, concluded HBx-SMYD3 interaction, guided by the downstream target gene c-myc, promoted cell proliferation [26]. Additionally, HBx-transgenic mouse models implicate enhanced expression of fibroblast growth factor-inducible 14 (fn14) in c-myc/TGF-α-driven hepatocarcinogenesis [27]. HBx also disrupts cell cycle progression by upregulating p21 and p27, proteins that inhibit cyclin-dependent kinase (CDK) activity, which in turn enhances the ability of mitogen-activated protein kinase (MAPK) signalling to cause proliferation in hepatocytes [28]. Similarly, HBx elevates serine/threonine p21 activated kinase 1 (PAK1) and causes cytoskeletal rearrangement in xenograft mouse models [29].

With regard to altering mitogenic signalling pathways, HBx promotes cell proliferation via the 5-LOX/FAS mediated positive feedback loop mechanism. In vitro studies in HepG2 and H7402 cells concluded that HBx upregulated the transcription of fatty acid synthase (FAS), known to play a critical role in tumour cell survival and proliferation, mediated by 5-lipoxygenase (5-LOX) through phosphorylated ERK 1/2 [30]. Another study also has found that HBx promotes liver cell division by upregulating cyclooxygenase (COX-2) and MERK/ERK kinase 2 (MEKK2), the latter known to regulate several transcription and translation factors [31]. COX-2, on the other hand, is highly expressed in HCC, and along with HBx, mediates sequestration of p53 to abolish apoptosis. Incapacitated p53 can induce reactivation of previously suppressed anti-apoptotic proteins such as Mcl-1 thus driving forward an indirect HBx-mediated cell proliferation [32]. HBV gene expression and hepatocyte transformation is also driven by Ras-Raf MAPK signalling pathway by HBx-mediated activation of Ras and Src kinase through overriding the pro-apoptotic effects of HBx which will be reviewed in the following sections [33,100]. Such signalling mechanisms are imperative for successfully stimulating cell cycle and deregulating cell cycle checkpoint controls [101].

HBx is also capable of enhancing cytosolic calcium levels leading to elevated mitochondrial calcium uptake which induces HBV replication in vitro [34]. HBx may be involved in extracellular matrix remodelling by elevating adhesion protein LASP-1 through PI3K pathway to promote hepatocyte proliferation and invasion [35]. It is worth noting that high concentrations of HBx and its subcellular localization, especially in cell systems that lack effective negative growth regulatory pathways and intact tumour suppressors, suggests that cell growth inhibition in such cells is not strictly governed to the extent of that of healthy cells, which could be a key driver of neoplastic transformation [102].

## 3. Evading Growth Suppressors

Neoplastic maladies arise from accumulated genetic and epigenetic changes in proto-oncogenes and tumour suppressor genes with the latter mostly involved in suppression of metastasis, pro-apoptosis and DNA damage repair [103]. For instance, mutations arising from the TP53 tumour suppressor gene have been implicated in over half of all human cancers [104]. HBx is known to bind to the C terminus of p53 and obstruct numerous crucial cellular processes such as transcriptional binding, DNA sequence-specific binding and apoptosis. More specifically, in human hepatocytes and fibroblasts, HBx partially sequesters p53 in the cytoplasm leading to unfavourable G1 arrest conditions that eventually result in inhibition of apoptosis, typically indicating onset of hepatocarcinogenesis [36,105]. Interestingly, transient transfection of human pulmonary adenocarcinoma Calu-6 cells has shown that HBx directly interacts with p53 and enables inhibition of p53 response element-directed transactivation [37].

Retinoblastoma-associated (Rb) tumour suppressor is another critical gatekeeper of cell cycle progression that is deregulated by HBx. It has been shown that HBx incapacitates inhibition of E2F1 activity, a positive regulator of cell cycle progression, by inactivating Rb gene promoter and thereby tumour suppressor Rb [38]. Additionally, HBx increases CDK2 activity, leading to impairment of E2F1–Rb balance, which confers stability to replication initiator protein CDC6 and aids in the HBx-mediated oncogenic sabotage thereafter [39].

MicroRNAs (miRNAs), which regulate gene expression through translational repression and degradation of complementary target mRNAs, define tumourigenesis [106]. miR-205, a miRNA tumour suppressor is inhibited by HBx via hypermethylation of miR-205 promoter [40]. Another vital tumour suppressor miR-520b which targets cyclin D1 and MEKK2 is known to inhibit liver cancer cell proliferation [107]. HBx stimulates hepatocarcinogenesis by partnering with survivin by controlling tumor suppressor miR-520b and oncoprotein hepatitis B X-interacting protein (HBXIP), binding protein of HBx [41]. Epigenetically, HBx also represses RIZ1, another tumour suppressor of HCC, via methytransferase 1 (DNMT1) governed hypermethylation and suppressed miR-152 [42]. Moreover, HBx acts as an epigenetic deregulator by altering transcription of DNMT1 and 3 and thereby suppresses E-cadherin tumour suppressor while hypermethylating p16 via pRb-E2f pathway [43,44]. Tumour-suppression activity of retinoic acid receptor-beta 2 (RAR-β2) is epigenetically downregulated by HBx via DNMT-driven hypermethylation, instigating upregulation of G1-checkpoint regulators p16, p21 and p27 and eventually E2F1 activation and ensued tumourigenesis [45,108].

## 4. Resisting Cell Death

Programmed cell death by apoptosis, initiated by highly regulated cascades of intrinsic and extrinsic pathways, is another hurdle tumour cells need to circumvent. Series of upstream regulators and downstream effectors of the apoptotic machinery regulate binding of Fas ligand on the cell membrane leading to activation of caspase 8 and caspase 3 and thereby extrinsic pathways, while the mitochondrial release of cytochrome c activates extrinsic pathways, leading to proteolysis that gradually dissembles the cells, eventually consumed by phagocytic neighbouring cells [17,109].

Intriguingly, HBx-induced apoptosis in HCC plays a contradictory role depending on the cellular conditions and components that HBx interacts with. For instance, pro-apoptotic HBx induces the expression of TRAIL-R2 (DR5), a death receptor that targets TNF µ-related apoptosis inducing ligand (TRAIL) that is known to elicit apoptosis [46]. HBx suppresses the E3 ubiquitin ligase activity of A20 via upregulation of miR-125a which prevents inhibition of caspase 8 leading to hepatocyte sensitization to TRAIL-induced apoptosis [47]. Conversely, anti-apoptotic HBx elevates the expression of widely known apoptosis inhibitor genes myeloid cell leukemia-1 (Mcl-1) and B cell lymphoma 2 (Bcl-2) which inherently restrains pro-apoptotic Bcl-2-associated X protein (Bax) further inactivating caspase 9 and 3 [48]. HBx also modulates apoptotic activities by acting on the mitochondria, caspases and SIRT-related pathways. More precisely, elevated cytoplasmic HBx interacts with apoptosis-inducing factor (AIF) and AIF-homologue mitochondrian-associated inducer of death (AMID) which primarily drives the inhibition of AIF translocation from mitochondrion-to-nucleus [49]. The discrepant behaviour of HBx-elicited apoptosis could be partly attributed to HBx mutations, such as C-terminal truncation (trHBx), which are significantly more common in tumour tissues compared to non-tumour tissues [110,111]. For example, expression of wild type HBx (wtHBx) impaired colony formation in several HCC cell lines while cell growth remained unaltered in cells transfected with trHBx, with some studies even suggesting that C-terminal transactivation might be the primary site of pro-apoptotic function [112]. However, it is worth noting that HBx intracellular localization could be another key driver of apoptosis, since wtHBx and trHBx displayed preferential localization in cytoplasm and nucleus, respectively [113,114].

Autophagy signifies another crucial cell-physiologic response that sequesters protein aggregates and impairs organelles into autophagosomes which fuse with lysosomes, resulting in degradation [50,115]. HBx is known to incapacitate lysosomal acidification and ensued decrease in lysosomal degradative capacity while simultaneously upregulating autophagy substrate SQSTM1 and lysosomal aspartic protease cathepsin D, which aids in chronic HBV infection followed by liver cancer [50]. Similarly, HBx interacts with BECN1 (Beclin 1) and hinders the interaction of BECN1-Bcl-2 complex that inhibits assembly of pre-autophagosomal components [51]. Contrariwise, In vitro studies in HepG2 cells suggest the progressive activation of autophagic lysosomal pathway by HBx via the PI3K-Akt-mTOR pathway [52]. Such paradoxical behaviour of HBx in driving autophagy of hepatocytes could be attributed to severely stressed cancer cells preferentially undergoing autophagy to achieve reversible dormancy [116]. Clarifying such conflicting behaviours of HBx-induced autophagic tumour cells could close a crucial research gap in liver cancer research.

## 5. Enabling Replicative Immortality

Contrary to the proliferative barriers that normal cells instil with limited number of division cycles, tumour cells display acquired unlimited replicative potential, propagated primarily by abrogating senescence and cell death. For instance, telomere maintenance is crucial to sustaining the neoplastic state. Healthy cells achieve apoptosis or senescence by decreasing telomerase activity and gradually shortening the telomeres, unlike tumour cells which preserve unaltered telomere lengths owing to persistent telomerase reactivation [117]. HBx is reported to impact on telomeres and telomerase activity; however, this remains controversial. A number of studies have suggested that HBx activates human telomerase reverse transcriptase (hTERT) mRNA expression in hepatocarcinoma and cholangiocarcinoma cells [53,54,118], whereas one study reported that HBx transgenic mice showed significantly low telomerase activity with virtually no difference in the mRNA expression of hTERT compared to healthy mice, during partial hepatectomy [119]. However, since there is high mutation rate in HBx, especially in different HBV genotypes, it is challenging to exclusively define telomerase activity in the HCC patient population as a whole. Canonically, one study argues that certain, not all, HBx isoforms are able to impair human telomerase expression by transcriptionally repressing its promoter, aided by myc-associated zinc finger protein (MAZ) binding to its consensus sequence. Nonetheless, telomere shortening is widely observed in hepatocarcinogenesis. They further speculate that HBx-mediated impaired telomerase activity could lead to genome instability and subsequent restoration of telomerase transcription that may aid in HCC cellular transformation [55].

## 6. Prompting Angiogenesis

Unregulated angiogenesis is a key hallmark of neoplastic growth and metastasis. Similar to normal tissues, tumour tissues too acquire nutrients and oxygen as well as dispel waste products by developing new blood vessels, known as angiogenesis. However, unlike the tightly regulated largely quiescent vasculogenesis in normal cells, cancer cells maintain a constantly activated “angiogenic switch” owing to several countervailing factors [120,121]. There is a strong correlation between HBV-induced angiogenesis and HCC progression and malignancy, especially by the only regulatory protein encoded by HBV: HBx [122].

Compelling evidence by Kleinman and Martin (2005) show that, in matrigel injected mice, HBx-expressing HCC cells induced more blood vessel formation compared to control HCC cells. Moreover, HBx alone could not only elicit angiogenesis but also upregulate VEGF mRNA expression as well as aid in transcriptional activation and stabilization of hypoxia-inducible factor (HIF-1α), the latter a key regulator of cellular adaptive responses to hypoxic conditions [56,57,58]. HBx directly interacts with transcriptional regulator bHLH/PAS domain of HIF-1α while mitigating binding of tumour suppressor protein von Hippel-Lindau (pVHL) and averting ubiquitin-dependent degradation of HIF-1α [58]. More specifically, HBx induces phosphorylation of HIF-1α while activating p42/44 mitogen-activated protein kinases (MAPK) [59]. HBx also drives HIF-1α-related angiogenesis by upregulating metastasis-associated protein 1 (MTA1), critical gene involved in tumour invasion and metastasis, and histone deacetylase (HDAC1) [60]. Increased expression of VEGF and HIF-1α has been observed in patient samples, thus entails a strong positive correlation with HBV-induced HCC [123,124]. HBx has been known to overexpress several matrix metalloproteinases (MMP 2 [61], 3 [62] and 9 [63]), which initiates neovascularization by disintegrating basement membrane thus ameliorating endothelial migration to nearby tissues. Moreover, HBx induction of COX-2 enzyme catalyses pro-angiogenic activity that further drives HBV-associated tumourigenesis [64].

Among key pathways that govern angiogenesis in general, Notch signalling is notable for its functions in cell development, wound healing, pregnancy and more importantly, tumour angiogenesis. More explicitly, Notch ligands Delta-like 4 (DII4) seem to act as a negative regulator of tumour angiogenesis. One study reported upregulated Notch1 and DII4 in HBV genome containing HepG2.2.15 cells compared to HepG2 control cells, also HBx silencing diminished DII4 levels and cleaved Notch1 [65]. Furthermore, specific signalling pathway inhibitor treatment assured that MEK1/2, PI3K/AKT and NF-κB pathways deem imperative for HBx-mediated Dll4 upregulation [65]. Angiogenesis in hypervascular tumours such as HCC is partly regulated by angiopoietins especially the well-characterized angiopoietin-1 (Ang1) and angiopoietin-2 (Ang2) which bind to the tyrosine kinase receptor. Unsurprisingly, HBx-expressing hepatocytes and HBV infected patient biopsies have shown significant stimulation of Ang-2 isoform expression [66]. Finally, successful vasculature and tumour invasion require endothelium-derived nitric oxide and according one study, HBx alone or HBV genome are capable of inducing nitrogen oxide synthase 2 (NOS2) mRNA expression; however, this potential mechanism warrants further research [67].

## 7. Triggering Invasion and Metastasis

Tumour metastatic dissemination and invasion is conceivably the most significant cancer hallmark, as all other hallmarks tend to be indistinctively represented in both benign and malignant tumours, unlike metastasis which, with the exception of endometriosis, tends to define malignancy for its unique characteristic ability to invade and colonize distant healthy tissues [125].

The influence of HBx on cell migration, invasion and extracellular matrix remodelling are largely associated with the upregulation of membrane-type matrix metalloproteinase (MMPs). Aggressive behaviour of HCC cells is partly driven by metastasis induced by HBx, which disrupts adherences junctions and integrin-associated adhesion to the extracellular matrix in addition to promoting production of MMPs 1, 3 and 9 [126]. HBx expressing cells have been shown to modify α integrin subunits and activate β1 integrin subunits which localize in pseudopods and prevents cell attachment, thus leading to epithelial transformation [69]. Calpain small subunit 1 (Capn4), a regulatory subunit involved in cell proliferation, migration and apoptosis, is highly correlated with tumour metastasis after liver transplantation [127]. Interestingly, HBx contributes to upregulation of Capn4 via nuclear factor-kB/p65 in HepG2 hepatoma cells [70]. Another study has also highlighted, HBx-mediated progression of tumour stemness in HCC via a positive feedback loop mechanism involving miR-5188 impairment of FOXO1 (a well-characterized tumour suppressor in HCC) and stimulation of β-catenin nuclear translocation [71]. Likewise, miRNA-driven HBx influence has been associated with metastatic behaviour, for instance, In vivo work by Zhang et al. demonstrates a dramatic elevation of miRNA-143 (miR-143) in HBx transgenic mice [72]. MiR-143 transcription is carried out by nuclear factor kappa B (Nf-Kb) which potentiates metastatic activities and fibronectin type III domain containing 3B (FNDC3B) which directs cell motility [72].

HBx alters migratory phenotype of transformed hepatocytes through cytoskeletal rearrangement and development of pseudopodial protrusions in addition to activating cell-surface adhesion molecule CD44, a hyaluronan (HA) receptor. This is achieved by HBx-mediated relocation of F-actin binding proteins (that belong to the ezrin/radixin/moesin family) in the pseudopodial protuberances, via a Rho/Rac- dependent manner while simultaneously enhancing CD44-moesin interaction [73]. While a majority of these studies focus on HBx wild type, one landmark study has concluded that HBx mutant, HBxΔ127 promotes hepatoma cell migration by activating ossteopontin (OPN), a secreted phosphoprotein well implicated in mammalian epithelial cell transformation, through 5-LOX (another key enzyme tied to metabolism of arachidonic acid and often upregulated in several tumor types) [74]. It is worth noting that HBV integration which most often leads to terminal truncations on HBx, was identified in 80%-90% of the host genome of HBV-induced HCC cases [128].

A key developmental regulatory process prominently involved in cancer metastasis and invasion is epithelial–mesenchymal transition (EMT). Among plethora of research focussing on EMT modulation by oncogenic viruses, HBV-induced HCC has been well implicated especially due to the high incidence of poor patient survival associated with metastasis in advanced tumour stages [129]. HBx ameliorates cancer motility and EMT by activating signal transducers and activators of transcription 5b (STAT5b) and c-Src proto-oncogene in hepatoma cells [75,76]. Moreover, HBx stabilizes Snail protein, a key EMT-orchestrating transcriptional factor, through triggering the phosphatidylinositol 3-kinase/protein kinase B/glycogen synthase kinase-3b (PI3K/AKT/GSK-3b) signalling pathway [77]. Lastly, HBx is known to induce the expression of vimentin, a putative mesenchymal marker, while epigenetically repressing E-cadherin, a key cell-to-cell adhesion molecule which best characterizes phenotypic alterations that are indicative of EMT-driven cellular transformation [78,79].

## 8. Evading Immune Destruction

Evading innate and adaptive immunity remains a crucial defensive mechanism that ensures HBV survival in infected hepatocytes. While it is expected for virus–host interactions to induce immune system activation, viruses with oncogenic potential, through evolution, have developed several strategies to surpass barriers of the immune system and establish chronic infections [130].

In particular, HBx imposes immune suppression by inducing apoptosis in HBV-specific CD8+ T cells (known to be major effector cells of viral clearance during acute HBV infection), while decreasing the production of interferon-ϒ in hepatocytes (a cytokine that suppresses HBV proliferation in hepatocytes) [80,131]. Beta interferon promoter stimulator 1 (IPS-1) is an adaptor protein that mediates retinoic acid-inducible gene 1 (RIG-1) signalling and is known to activate antiviral innate immune response and interferon β (IFN-β). HBx-IPS-1 protein interaction interferes with RIG-1-related immuneregulation and inhibits activation of IFN-β [81]. Similarly, HBx mediates inhibition of IFN-regulatory factor (IRF3) and disrupts associations of the mitochondrial membrane protein virus-induced signalling receptor (VISA) with its upstream components RIG-1 and melanoma differentiation-associated gene 5 (MDA5) [82]. Another HBx/IFN-β linked immune suppression mechanism is the interaction between HBx and mitochondrial antiviral signalling (MAVS) protein, a critical activator of Nf-kB and IRF-3, and degradation of MAVS via Lys-136 ubiquitination that prevents IFN-β induction [83]. Moreover, HBx protein impairs glucose metabolism with augmented lactate production, which weakens the migratory ability of T cells in the liver as well as their cytolytic activity [132]. Additionally, HBV evades immune recognition with the aid of HBx which transcriptionally promotes RNA adenosine deaminase ADAR1 resulting in HBV RNA deamination of adenosine (A) to generate inosine (I),which disrupts host immune recognition in hepatocytes [84].

Attenuation of the antiviral immune response can also be accomplished by HBx-mediated epigenetic modulation and miRNA dysregulation [133]. HBx-dysregulated tumour suppressor miR-101 reduces LPS-stimulated macrophage expression, capable of inducing HCC, due to its inability to modulate DUSP1 phosphatase enzyme, which leads to impaired ERK1/2/p38/JNK elevation of pro-inflammatory cytokines such as TGF-β [134]. To gain a more comprehensive insight into HBx influence on a plethora of miRNA dysregulation pathways and their associated immune response, refer to Sartorius et al. [133].

## 9. Tumour-Promoting Inflammation

Inflammation, an adaptive response to infection and tissue damage, paradoxically contributes towards tumourigenesis via facilitating other cancer hallmarks by delivering bioactive molecules to the tumour microenvironment, such as anti-apoptotic survival factor, growth factors, angiogenesis-driving extracellular matrix-modifying enzymes, proangiogenic factors and inductive signals that aid in cellular transformation [22]. Non-resolving inflammation which substantially contributes to HCC, is initiated by extrinsic pathways involving pattern-recognition receptors (PRRs) by pathogen-associated molecule patterns (PAMPs) resulting from gut microflora or damage-associated molecule patterns (DAMPs) released from apoptotic liver cells. Such inflammation-mediating DAMPs include nuclear and cytoplasmic proteins, such as, histones and IL-1, high-mobility group box1 (HMGB1), heat shock proteins and myeloid-specific S100 proteins (S100s). Similarly, intrinsic pathways, including the recruitment of inflammatory cells by the tumour itself, too contribute to HCC disease pathogenesis [135].

HBx overexpression in healthy hepatocytes induces serine/threonine kinases, in particular, receptor-interacting protein (RIP-1), which inherently aids in the production of pro-inflammatory cytokines IL-6, IL-8 and CXCL2, in addition to secreting HMGB1, a cytokine mediator of inflammation [85]. Moreover, HBx has been mechanistically demonstrated to induce S100A9 DAMP protein, by prompting translocation of cytoplasmic NF-kB into the nucleus followed by binding to activate its transcription [86]. Activated S100A9 binds to PRR receptor RAGE in the tumour microenvironment and blocks host-mediated antitumor immune response targeting tumour cells, in doing so enabling tumour progression [136,137].

HBx induces immunomodulation by upregulating inflammatory cytokines and other related cellular components such as ICAM-1, major histocompatibility complex and Fas ligand, thus promoting long term liver inflammation and ensuing HCC [138,139]. Paradoxically, HBx may also hinder HBV-specific immune response, promote apoptosis of immune cells and induce immune response leading to persistent HBV infection and liver tumourigenesis [140]. In vitro, HBx stimulates production of interleukin-6 (IL-6) while activating downstream signalling targets of innate immune signal transduction adaptor MyD88, including, IRAK-1, NF-kB and ERKs/p38 in the HBV-infected liver microenvironment [87]. Besides, chemokine expression profiles in hepatoma cells show that IL-8 is significantly upregulated when HBx is overexpressed, while downregulation was observed in pro-inflammatory mediators such as, macrophage inflammatory protein-3β (MIP-3β), epithelial-neutrophil activating protein 78 (ENA78) and monocyte chemotactic proteins-1, -2 and -3 [141]. In chronic HBV patients, HBx also invokes activation of ERK/NF-kB pathway which leads to transactivation of the p19 and p40 subunits of IL-23, predominantly secreted by macrophages and dendritic cells which are mainly activated by proinflammatory cytokines [88]. Another study reports that HBx-mediated tumour microenvironment remodelling involves interactions with centrosomal P4.1-associated protein (CPAP), a key regulator of procentriole elongation and microtubule nucleation, which progressively leads to enhanced inflammatory cytokine production, malignancy associated with NF-kB activation [89].

## 10. Genome Instability and Mutation

Successful establishment of neoplasms and eventual dominance over the tumour microenvironment, in part require alterations in the premalignant cell genome and amassing favourable genotypes. Tumour cells hijack the host DNA-maintenance machinery by damaging the host cell’s ability to detect and respond to DNA damage, activate DNA repairing and inactivate mutagenic molecules [22]. HBV in known to productively infect hepatocytes by taking over DNA damage response proteins and indirectly influencing intracellular signalling pathways that determine DNA repair [142].

HBx induces genetic instability and defects in chromosomal segregation partly by binding with the conserved HBx-interacting protein (HBXIP) and driving aneuploidy associated with overabundant centrosome formation as well as tripolar and multipolar mitotic spindles. HBx promotes aneuploidy, a classic trait of tumourigenicity, by disrupting the cell growth stimulating function of HBXIP, resulting in failed centrosome duplication during prometaphase. HBXIP and HBx prevent the dissociation of midbody microtubules that hold the dividing daughter cells together during telophase, which leads to merged binucleated cells [90]. Additionally, HBx has been shown to contain a leucine-rich nuclear export signal (NES) domain which binds to nuclear export receptor Crm1 and activates NF-kB nuclear translocation. Given Crm1 plays a role in mitosis, HBx-mediated inactivation of Crm1 via its nuclear export could be a key affair that drives HBV related oncogenesis [91]. Additionally, since Crm1 is involved in maintaining centrosome integrity, disrupting Crm1 activity by HBx indirectly drives formation of supernumerary centrosomes and abnormal multipolar spindles [143]. HBx overexpression in healthy hepatocytes displays synthesis of polyploidy nuclei, DNA damage accumulated via elevated IL-6 expression and PLK1 and p38/ERK activation which strongly correlates with aberrant polyploidation and hepatocyte transformation [144].

With regard to HBx-mediated disruption of DNA repair mechanisms, HBx binds with damaged DNA binding (DDB) proteins causing alterations in p53 function, which in turn induces cell apoptosis and inhibits nucleotide excision repair (NER), thereby compromising genome integrity [92,145]. Furthermore, Ras-induced senescence is interrupted by HBx leaving hepatocytes cells vulnerable to mitotic errors, caused by supernumeracy centrosomes and multipolar spindles [93]. HBx also stimulates DNA helicase catalytic activity of TFIIH subunits while recruiting DNA-repair enzyme tyrosyl-DNA phosphodiesterase 2 (TDP2) for the synthesis of HBV DNA [94,146]. Chromosome segregation and organization are crucial functions of the structural maintenance of chromosomes (Smc) complex proteins, Smc 5/6 in particular. Relevantly, given the multi-regulatory functions of HBx, it is not surprising that HBx promotes host cell genetic instability via degradation of Smc 5/6 proteins following binding with cellular DDB1-containing E3 ligase [95,147].

## 11. Deregulating Cellular Energetics

Altered energy metabolism is another crucial malignancy trait that is closely associated with exponential proliferation-inducing hallmark of cancer cells, with regard to the abnormal Warburg effect of cancer cell metabolism. One study shows that C-terminal truncated HBx in capable of inducing neoplasmic traits by downregulating an important regulator of glucose sensing and reduction–oxidation system, thioredoxin-interacting protein (TXNIP), which drives glucose metabolism reprogramming [96]. This finding remains significant as TXNIP has been implicated as a tumour suppressor and its expression is either low or redundant in hepatoma cells [148]. Cancer cells promote their aerobic glycolysis-driven energy metabolism by upregulating glucose transporters such as GLUT1, to increase glucose uptake into the cytoplasm. Oncogene HBx interacting protein HBXIP elevates GLUT1 via upregulating NF-kB in liver cancer [97]. Another recent study has concluded that hepatic glucose homeostasis is retained by HBx via elevating the gene expression of hepatic gluconeogenic enzymes, namely, PEPCK, PGC1α, and G6Pase and glucose production which are inherently regulated through nitric oxide (NO)/JNK signalling [98].

HBx contributes to abnormal energy metabolisms in hepatic cells by promoting lipid peroxidation and reactive oxygen species (ROS). In hepatoma cell lines, HBx also reduces expression of mitochondrial enzymes that are part of electron transport in oxidative phosphorylation (complexes I, III, IV, and V) while sensitizing the mitochondrial membrane potential [149]. HBx induces oxidative stress and mitochondrial injury by downregulating NADPH: quinone oxidoreductase 1 (NQO1) enzyme detoxifies ROS, thereby indirectly inducing glycolysis that drives carcinogenesis. Interestingly, C-terminal truncated HBx-induced mitochondrial damage is more severe than nucleus DNA [99].

## 12. Therapeutic Potential of HBx

Since HBx expression persists throughout from the inception of acute HBV infection to the progression of HCC, spanning a couple of decades of repeated cycles of chronic liver inflammation, it could be considered an ideal therapeutic target. While research into the potential therapeutic implications of HBx is still in its infancy, identifying HBx-interacting host cell targets and better understanding HBx-mediated host immune response, could potentially help reverse the chronic inflammatory state during HBV, and thereby preventing liver cellular transformation.

However, given the multi-regulatory functions of HBx, especially its pervasive role in every cancer hallmark, it remains challenging to narrow down the search for prospective therapies [150]. Another limitation is that due to the difficulty in obtaining appropriate protein samples, the native full-length polypeptide sequence of HBx has not been deciphered by NMR or x-ray structural characterization [151]. Additionally, standardized protocols are yet to be established for studying HBx [152]. Even though the majority of the HBx-related studies, as detailed in this review, rely on HBx-overexpression In vitro studies, these models fall short of deciphering its real physiological relevance. Another limitation can be attributed to the double-edged nature of HBx, for instance, its contradictory role in both promoting and inhibiting apoptosis as well as alternating between activating and hindering host immune response, throughout the course of the HBV infection.

Despite such shortcomings, experimental progress in HBx-based therapeutics are currently being pursued worldwide. Recently, HBx protein-based therapeutic vaccine has been shown to drive HBV viral antigen clearance by mobilizing systemic HBx-mediated CD4+ and CD8+ T cell response in HBV carrier mice [153]. Similarly, In vitro and In vivo HBx-knockdown models using short hairpin or short interfering RNA have been shown to abrogate HBx activity and promote antiviral response and anti-tumourigenicity [154,155]. HBx monoclonal antibody shows promise as an anti-tumour agent based on HCC mouse models and clinical studies. HBx antibody bound cell penetrating HIV tat protein which is conjugated experimentally to enhance antibody delivery to the cell, inhibits HBV transcription and translation. Combinatorial therapies utilizing HBx antibody and HBV capsid inhibitors that eliminate viral infection could aid in long term containment of chronic HBV infection and tumour emergence [156,157]. Alternatively, more research needs to be focused into repurposing drugs currently existing in the market. For instance, Dicoumarol, a competitive NQO1 inhibitor, has been verified as an inhibitor of HBx expression, displaying strong antiviral activity in humanised mouse model [157]. Similarly, FDA-approved Nitazoxanide, repurposed from its prescription for protozoan enteritis, inhibits the HBx-DDB1 protein interaction and restores Smc5 protein expression and prevents viral transcription and translation [158]. Another study which conducted large-scale screening of 640 FDA-approved drugs against HBV, concluded that a few of these compounds (24 most potent compounds) decreased HBV transcription by, on average, 33.9% in the absence of HBx expression and 30.6% in the presence of HBx. Further analysis showed one compound, Terbinafine, potently and specifically impaired HBx-mediated HBV RNA transcription In vitro [159].

More precisely, among principal clinical trials in randomized Phase II trial evaluating antiviral therapies to combat HBV, the recombinant yeast-based vaccine, GS-4774, which contains HBx, HBsAg and HBcAg viral components capable of eliciting an immune response by promoting antigen processing via MHC class I and II pathways, was assessed. The study concluded that, while the vaccine was well-tolerated, there was limited efficacy observed, with no significant clinical outcomes in HBsAg clearance, in virally suppressed non-cirrhotic patients with chronic HBV infection [160]. Similarly, another randomized Phase II trial using combinatorial therapy of GS-4774 and tenofovir disoproxil fumarate (TDF), showed clear restoration of several T-cell functions in HBeAg negative patients with hepatitis B even though the therapy fell short on producing clinically significant decrease in HBsAg in these patients [161,162]. Notably, therapies solely based on HBx are yet to reach clinical trials. Nonetheless, these promising observations further emphasize the significance of targeting HBx along with combinatorial therapies in eliminating HBV-induced hepatocellular carcinoma.

## 13. Conclusions and Future Prospects

The high mortality rate associated with HBV-induced hepatocarcinogenesis has encouraged intense research into better understanding the cellular and physiological mechanisms of HBV-induced liver malignancies. With regard to HBx viral protein, its multifactorial nature prevents current research from identifying a specific function of HBx that potentially drives cellular transformation. Unsurprisingly, it is even more challenging to identify suitable cellular targets for HBx-based therapy since it is difficult to quantify the cellular and nuclear localization of HBx at a particular point over the course of the HBV infection, as this is subjected to frequent alterations depending on the HBV viral load, tumour microenvironment and other risk factors the liver encounters (hepatitis C, metabolic diseases, chronic alcoholism and aflatoxin exposure) [163]. Nonetheless, the past few decades have witnessed a surge in research focussing on the role of HBx in the initiation, progression and metastasis of HCC.

While decades of research have suggested the potential role of HBx from genetic and epigenetic standpoints, especially its implication on HCC pathogenesis, the emerging field of epitranscriptomics, the study of RNA modifications, is an untrodden territory that warrants research. Few studies have suggested certain RNA modifications such as m6A to play a crucial role in HCC, yet the role of the HBx protein in this context remains unclear, highlighting a research gap with immense therapeutic prospective [164,165,166,167,168,169]. It is also worth exploring the mechanisms HBx employs to control the level of HBV replication and which exact domains and host cell targets are modulated by HBx and their concomitant biological relevance. A better understanding how HBx regulates various hallmarks of HCC would provide rather an overall picture of HBV-mediated HCC. In this context, it is imperative to take into account the short half-life and unstructured nature of HBx when designing experimental models to closely emulate, as much as possible, actual liver disease pathogenesis. Thus, elucidating the role of HBx in hepatocarcinogenesis could be the key to unlocking promising therapeutic strategies to combat HCC.

## Figures and Tables

**Table 1:** Key molecular mechanisms regulated by hepatitis B Virus X protein that drive hallmarks of hepatocellular carcinoma.

HCC Hallmark	HBx Activity	Study Design	Ref.
Sustaining proliferative signalling	Activates stellate cells and elevates transforming-growth factor β1 (TGF-β1) and connective tissue growth factor (CTGF)	In vitro co-culture with LX-2 cells and stable QSG7701-HBx cell line	Guo et al. [23]
	Five-fold elevated expression of c-myc	In vitro human hepatoma cell lines Huh7 and IHHs and in vivo X15–myc transgenic mouse model In vivo HBx-transgenic mice with c-myc driven by woodchuck hepatitis virus (WHV)	Shukla & Kumar et al. Terradillos et al. [24,25]
	HBx-SMYD3 interaction, guided by the downstream target gene c-myc	In vitro HBx-expressing HepG2 cells and HBV containing HepG2.2.15 cells	Yang et al. [26]
	Enhanced expression of fibroblast growth factor-inducible 14 (fn14)	In vitro human fibroblasts and HCC cells and In vivo HBx-transgenic mice.	Feng et al. [27]
	Disrupts cell cycle progression by upregulating p21 and p27	In vivo pX expressing primary mouse hepatocytes	Qiao et al. [28]
	Elevates serine/threonine p21 activated kinase 1 (PAK1)	In vivo tumour xenografts in mice and in vitro human hepatoma cells with pHBV1.3	Xu et al. [29]
	Upregulates transcription of fatty acid synthase (FAS), mediated by 5-lipoxygenase (5-LOX)	In vitro HBx-expressing human hepatoma HepG2 and H7402 cells	Wang et al. [30]
	Upregulates cyclooxygenase (COX-2) and MERK/ERK kinase 2 (MEKK2)	In vitro HBx-expressing L-O2 and H7402 cell lines.	Shan et al. [31]
	Suppressed anti-apoptotic protein Mcl-1	In vitro Chang liver cells transiently transfected with HBx (CHL-X)	Lee et al. [32]
	Activates Ras and Src kinase	In vitro human hepatoma Hep3B cells with transiently transfected HA-tagged HBx	Noh et al. [33]
	Enhances cytosolic calcium levels	In vitro HepG2 cells transfected with full length HBx	Yang & Bouchard, 2012 [34]
	Elevates adhesion protein LASP-1 via PI3K pathway	In vitro HBx stably transfected HepG2 and Huh-7 cells	Tang et al. [35]
Eluding growth suppressors	Partial sequestration of p53 causing G1 arrest	In vitro HBx expressing human fibroblasts, HepG2 cells and liver tissue from patients	Elmore et al. [36]
	Inhibition of p53 response element	In vitro HBx transiently transfected human Calu-6 cells	Truant et al. [37]
	Inactivates Rb gene promoter	In vitro HepG2 and Hela cells	Choi et al. [38]
	Confers stability to replication initiator CDC6	In vitro HBx-expressing human hepatoma cells Huh7, HepG2. In vivo X15-myc transgenic mouse model	Pandey & Kumar, 2012 [39]
	miR-205 inhibition via promoter hypermethylation	In vitro HBx-expressing hepatoma cell lines and In vivo HBx- transgenic mice and patient samples	Zhang et al. [40]
	Controls miR-520b and hepatitis B X-interacting protein (HBXIP)	In vitro HBx-expressing human hepatoma cells and In vivo nude mice transplantation and patient samples	Zhang et al. [41]
	Represses RIZ1 via hypermethylation	In vitro HBx-expressing human hepatoma cells and patient samples	Zhao et al. [42]
	Suppresses E-cadherin tumour suppressor	In vitro HBx-expressing HepG2 cell line	Lee et al. [43]
	Hypermethylates p16 via pRb-E2f pathway	HBV-HCC patient and tissue specimens	Zhu et al. [44]
	Downregulates retinoic acid receptor-beta 2 (RAR-β2)	In vitro HBx-expressing HepG2 cells	Jung et al. [45]
Resisting cell death	Pro-apoptosis		
	Induces the expression of TRAIL-R2 (DR5)	In vitro HBx-expressing Huh-7 cells	Kong et al. [46]
	Upregulation of miR-125a	In vitro HBx-expressing HepG2 and LO-2 liver cells	Zhang et al. [47]
	Anti-apoptosis		
	Induces myeloid cell leukemia-1 (Mcl-1) and B cell lymphoma 2 (Bcl-2)	In vitro HBx-expressing HPCs (HP14.5) cells	Shen et al. [48]
	Interacts with apoptosis-inducing factor (AIF) and AIF-homologue mitochondrian-associated inducer of death (AMID)	In vitro HBx-expressing HepG2 cells	Liu et al. [49]
	Autophagy		
	Upregulating SQSTM1 and lysosomal aspartic protease cathepsin D	In vitro HBx-expressing Huh-7 cells and human tissue specimens	Liu et al. [50]
	Interacts with BECN1 (Beclin 1)	In vitro HBx-expressing HepG2 and SK-Hep-1	Son et al. [51]
	PI3K-Akt-mTOR pathway	In vitro HBx-expressing HepG2 cells	Wang et al. [52]
Facilitating replicative immortality	Activates human telomerase reverse transcriptase (hTERT)	In vitro HBx-expressing HepG2 and QBC939 cell lines	Zhang et al. Qu et al. [53,54]
	MAZ binding aided telomerase impairment	In vitro HBx-expressing H7402 hepatoma cells	Su et al. [55]
Prompting angiogenesis	Upregulates VEGF mRNA expression and stabilizes HIF-1α	In vitro HBx-expressing human HepG2 and mouse Hepa 1–6 HCC cell lines In vitro ChangX-34 and HBx transgenic mice model In vitro HBx-expressing HEK293 cells	Lee et al. Moon et al. Yun et al. [56,57,58]
	Mitigates binding of von Hippel-Lindau (pVHL)	In vitro HBx-expressing HEK293 cells	Moon et al. [58]
	Activates p42/44 mitogen-activated protein kinases (MAPK)	In vitro HBx-expressing human hepatoma cell lines and HBx transgenic mice model	Yoo et al. [59]
	Upregulates metastasis-associated protein 1 (MTA1) and histone deacetylase (HDAC1)	In vitro Chang X-34 cells, HBx transgenic mice and patient samples	Yoo et al. [60]
	Overexpresses matrix metalloproteinases (MMP) 2,3 and 9	In vitro HBx-expressing human Chang cell lines and murine AML-12 liver cell line In vitro HBx-expressing human hepatoma cell lines In vitro HBx-expressing HepG2 cell line and xenograft mice model	Lara-Pezzi et al. [61] Yu et al. [62] Liu et al. [63]
	Induces COX-2 enzyme	In vitro HBx-expressing Hep3B cell line and patient samples	Cheng et al. [64]
	Mediates Dll4 upregulation	In vitro HBx-expressing human hepatoma cell lines and HCC patient samples	Kongkavitoon et al. [65]
	Stimulates Ang-2 isoform	In vitro HBx-expressing Chang cell line and rat hepatic stellate cells CFSC-2G, THP1 promonocyte cell line and patient and tissue specimens	Sanz-Cameno et al. [66]
	Induces nitrogen oxide synthase 2 (NOS2)	In vitro HBV-expressing HepG2 and HepG2.2.15 cell lines and patient and tissue specimens	Majano et al. [67]
Triggering invasion and metastasis	Promotes production of MMPs 1 and 2 and disrupts adherens junctions	In vitro HBx-expressing human Chang cell lines and murine AML-12 liver cell line HCC patient tissue specimens	Lara-Pezzi et al. [61] Giannelli et al. [68]
	Modifies α integrin subunits and activates β1 integrin subunits	In vitro HBx-expressing Chang cell line	Lara-Pezzi et al. [69]
	Upregulation of Capn4 via nuclear factor-kB/p65	In vitro HBx-expressing HepG2 and H7402 cell lines	Zhang et al. [70]
	Promotes tumour stemness via impaired FOXO1 and β-catenin nuclear translocation	In vitro HBx-expressing cell lines and In vivo tumour xenograft mice model	Lin et al. [71]
	Elevates miRNA-143 (miR-143)	In vitro HepG2 and Huh7 cell lines and In vivo HBx transgenic mice and patient tissue samples	Zhang et al. [72]
	Activates cell-surface adhesion molecule CD44	In vitro HBx expressing Chang cell line	Lara-Pezzi et al. [73]
	Activates ossteopontin (OPN) through 5-LOX	In vitro HBx-expressing HepG2 cell line	Zhang et al. [74]
	Activates (STAT5b) and c-Src proto-oncogene	In vitro HBx-expressing Huh7 and HCC patient samples In vitro HBx-expressing SMMC-7721 cell line	Lee et al. [75] Yang et al. [76]
	Stabilizes Snail protein	In vitro human hepatoma Huh7 and Chang cell lines and patient samples	Liu et al. [77]
	Induces expression of vimentin	In vitro HBx-expressing HepG2 and Huh7 cell lines and patient samples	You et al. [78]
	Represses E-cadherin	In vitro HBx-expressing HepG2 cell line and patient samples	Arzumanyan et al. [79]
Evading immune destruction	Induces apoptosis in HBV-specific CD8+ T cells	In vitro HBx-expressing primary hepatocytes	Lee et al. [80]
	Interacts with IPS-1 and inhibits interferon-ϒ	In vitro HBx-expressing HepG2 and In vivo HBx transgenic mice	Kumar et al. [81]
	Inhibits IRF3 and associations between VISA and RIG-1/MDA5	In vitro BHK and HEK 293 cell lines	Wang et al. [82]
	Degradation of MAVS via Lys(136) ubiquitination	In vitro human hepatoma cell lines, In vivo HBx knock-in mice model and liver tumour samples	Wei et al. [83]
	Promotes RNA adenosine deaminase ADAR1	In vitro HepG2.2.15 and NTCP-expressing HepG2 and Huh7, In vivo mice model	Wang et al. [84]
Tumour-promoting inflammation	Induces RIP-1 and aids in activation of cytokines IL-6, IL-8 and CXCL2	In vitro HBx-expressing LO-2 hepatocytes	Xie & Huang [85]
	Induces S100A9 DAMP protein	In vitro HBx-expressing human hepatoma cell lines, In vivo HBx transgenic mice model and patient samples	Duan et al. [86]
	Activates signal transduction adaptor MyD88, including, IRAK-1, NF-kB and ERKs/p38	In vitro HBx-expressing human hepatic L02 cells and human hepatoma SMMC-7721	Xiang et al. [87]
	Activates ERK/NF-kB pathway and IL-23 subunits	In vitro HepG2 and Huh7, normal hepatocyte Chang liver and HL-7702, and HepG2.2.15 cells lines and patient samples	Xia et al. [88]
	Interacts with CPAP regulator	In vitro HBx- and NTCP-expressing human hepatoma cell lines and In vivo xenograft mice model	Yen et al. [89]
Inducing genomic instability	Binds HBXIP	In vitro Hela and mouse embryonic fibroblast (MEF) cell lines and In vivo liver regeneration mice model	Fujii et al. [90]
	Binds Crm1 with the NES domain on HBx	In vitro HBx-expressing Hep3B and primary human fibroblast cell lines	Forgues et al. [91]
	Binds DDB proteins	In vitro wild-type or mutant HBx-expressing HepG2 cell lines	Becker et al. [92]
	Disrupts Ras-induced senescence	In vitro HBx-expressing human primary fibroblasts BJ and TIG3 cell lines and In vivo mice model	Oishi et al. [93]
	Stimulates DNA helicase catalytic activity of TFIIH subunits	In vitro HBx-expressing Hela cells and yeast model	Qadri et al. [94]
	Degradation of Smc 5/6	In vitro wild type and mutant HBx-expressing HepG2, HepAD38, HepG2-NTCP cell lines	Murphy et al. [95]
Deregulating cellular energetics	Downregulates TXNIP protein	In vitro HBx-expressing MIHA and LO-2 cell lines, In vivo mice model and HCC patient samples	Zhang et al. [96]
	Elevates expression of GLUT1	In vitro human hepatoma HepG2 cell line and HCC patient samples	Zhou et al. [97]
	Overexpression of PEPCK, PGC1α, and G6Pase	In vitro HBx-expressing HepG2 cell line, In vivo HBx transgenic mice and HCC patient samples	Shin et al. [98]
	Downregulates NQO1 enzyme	In vitro HBx-expressing Huh7 cell line	Jung et al. [99]
